# Integrated management of acute pulmonary embolism and the post-pulmonary embolism syndrome: risk stratification, modern therapies, and long-term follow-up

**DOI:** 10.3389/fcvm.2026.2002611

**Published:** 2026-10-21

**Authors:** Maria-Laura Craciun, Cristiana Adina Avram, Ana-Maria Pah, Simina Crisan, Diana-Maria Mateescu, Ioana Cotet, Cristina Vacarescu, Oana Patru, Maria Rada

**Affiliations:** 1 Department VI, Cardiology, Faculty of Medicine, “Victor Babeș” University of Medicine and Pharmacy Timișoara, Timișoara, Romania; 2 Department of Internal Medicine I, Faculty of Medicine, “Victor Babeș” University of Medicine and Pharmacy Timișoara, Timișoara, Romania; 3 Department of Infectious Diseases, Municipal Emergency Clinical Hospital Timișoara, Timișoara, Romania; 4 Doctoral School, “Victor Babeș” University of Medicine and Pharmacy Timișoara, Timișoara, Romania

**Keywords:** anticoagulation, catheter-directed intervention, chronic thromboembolic pulmonary hypertension, post-pulmonary embolism syndrome, pulmonary embolism, risk stratification, thrombolysis, venous thromboembolism

## Abstract

Pulmonary embolism (PE) is increasingly recognized as an episode of care that extends well beyond hospital discharge. Current guidance is distributed across several documents: the 2019 European Society of Cardiology (ESC) guidelines; the 2026 American Heart Association/American College of Cardiology (AHA/ACC) multisociety guideline, which replaced the massive/submassive terminology with clinical categories A–E; and separate position papers on catheter-directed intervention (CDI) and on follow-up. This narrative review (i) provides a side-by-side comparison of the ESC and AHA/ACC risk frameworks and their therapeutic implications; (ii) updates the evidence on reperfusion, including the recent randomized trials HI-PEITHO, PEERLESS and STORM-PE; and (iii) focuses on transitions of care and long-term follow-up, which guidelines address less comprehensively. Anticoagulation, preferably with a direct oral anticoagulant, remains the treatment for most patients. Systemic thrombolysis is reserved for high-risk (ESC) or category E (AHA/ACC) PE, because in intermediate-risk disease it halves hemodynamic decompensation at the cost of a several-fold increase in major and intracranial bleeding, without long-term functional benefit. In HI-PEITHO, ultrasound-facilitated catheter-directed thrombolysis reduced a 7-day composite outcome driven by cardiorespiratory decompensation (4.0% vs. 10.3%) in intermediate-high-risk patients with additional markers of physiological stress, without intracranial hemorrhage; a survival benefit of CDI has not been demonstrated. At discharge, a minimum bundle should specify the drug, dose, anticipated duration, provoking context, bleeding mitigation and follow-up ownership, followed by early contact within one week and a structured review at approximately three months. Up to half of survivors report persistent dyspnea or exercise limitation, whereas chronic thromboembolic pulmonary hypertension (CTEPH) develops in approximately 2%–3%. Persistent symptoms should prompt phenotype-directed evaluation with echocardiography, ventilation/perfusion scintigraphy, cardiopulmonary exercise testing and, when indicated, right-heart catheterization interpreted with the 2022 hemodynamic definitions, followed by early referral to an expert center where pulmonary endarterectomy, balloon pulmonary angioplasty and medical therapy can be combined. We state the type of evidence underpinning each key recommendation, propose a discharge bundle, a structured follow-up algorithm and episode-wide quality indicators, and identify the evidence gaps that should guide future trials.

## 1 Introduction

Acute pulmonary embolism (PE) is the third most frequent acute cardiovascular syndrome after myocardial infarction and stroke, with an annual incidence of approximately 39–115 per 100,000 population (1). Although age-standardized PE-related mortality in the European Union approximately halved between 2000 and 2015 (2), PE remains associated with early death, recurrent venous thromboembolism (VTE), treatment-related bleeding and substantial post-acute morbidity (1, 3, 4).

The care gap increasingly lies after initial survival. Up to half of patients report persistent dyspnea, reduced exercise capacity or impaired quality of life months after the index event (5, 6), and in the prospective FOCUS cohort the two-year cumulative incidence of post-PE impairment was 16.0% (7). Only a minority develop chronic thromboembolic pulmonary hypertension (CTEPH), but distinguishing deconditioning, comorbidity, persistent right ventricular (RV) dysfunction, chronic thromboembolic pulmonary disease (CTEPD) and CTEPH requires a structured pathway, and fragmented follow-up delays the diagnosis of operable disease (4, 8).

Several authoritative documents already address parts of this pathway (Table 1). The 2019 European Society of Cardiology (ESC) guidelines (1) and the 2026 American Heart Association/American College of Cardiology (AHA/ACC) multisociety guideline (9) cover diagnosis, risk stratification and acute treatment; the 2022 ESC position paper on follow-up (4) and the European Respiratory Society (ERS) statement on CTEPH (8) cover the post-acute phase; the 2022 ESC/ERS pulmonary hypertension guidelines define the hemodynamic thresholds (10); and recent consensus documents address catheter-directed interventions (11, 12). The 2026 AHA/ACC guideline itself spans diagnosis through follow-up, but these documents were developed in parallel and use different terminology—most visibly the ESC four-tier risk model versus the AHA/ACC clinical categories A–E—and a systematic comparison of international guidelines has identified areas of harmony, dissonance and silence, including the absence of a universal definition of shock in PE (13). The contribution of this review is therefore not to restate guideline recommendations, but to provide three elements that are not available in any single document: (i) an explicit side-by-side comparison of the European and American risk frameworks, with particular attention to the clinical implications of the new categories C–E, including incipient cardiopulmonary failure and normotensive shock; (ii) an evidence-graded update of acute reperfusion, incorporating the randomized trials of catheter-directed intervention (CDI) published since 2022 and stating the type of evidence that supports each key recommendation; and (iii) an operational framework linking acute decisions to longitudinal follow-up, consisting of a minimum discharge bundle with an acute-phase handover dataset, a phenotype-directed post-PE pathway and episode-wide quality indicators. The integrated pathway is summarized in the central illustration (Figure 1).

**Table 1:** Positioning of this review relative to existing guidance documents.

Document	Main scope	Coverage of transitions of care and follow-up	What this review adds
2019 ESC guidelines on acute PE (1)	Diagnosis, ESC four-tier risk model, acute treatment, duration	Brief section; follow-up at 3–6 months; referral of symptomatic patients with mismatched defects	Comparison with AHA/ACC categories; post-2019 CDI trials; operational discharge bundle
2026 AHA/ACC multisociety guideline (9)	Clinical categories A–E, acute treatment including CDI, follow-up	1-week contact, visit ≤3 months, symptom enquiry for ≥1 year, PE clinics	Side-by-side mapping to ESC classes; practical integration with European follow-up algorithms
2022 ESC position paper on follow-up (4)	Follow-up after acute PE, post-PE syndrome, CTEPD/CTEPH	Comprehensive; algorithm at 3–6 months	Link of follow-up triggers to acute-phase data; update with InShape II, FOCUS, rehabilitation RCTs, CTEPH therapy
ERS statement on CTEPH 2021 (8) and 2022 ESC/ERS PH guidelines (10)	Definitions and management of CTEPD/CTEPH; hemodynamic thresholds	Starts at suspicion of chronic disease	Translation of the 2022 thresholds into the post-PE pathway
European expert Delphi consensus on follow-up 2025 (85)	Key elements of follow-up focusing on CTEPH, CTEPD and post-PE syndrome	Consensus on timing, tests, setting and uniform protocol	Integration with acute-phase handover data and screening algorithms (InShape II/IV) into one pathway
ESC consensus on percutaneous treatment 2022 (11); SEC position statement 2025 (12)	Indications and techniques of CDI	Not addressed	Updated synthesis including HI-PEITHO, PEERLESS and STORM-PE; consistent CDI terminology

AHA/ACC, American Heart Association/American College of Cardiology; CDI, catheter-directed intervention; CTEPD, chronic thromboembolic pulmonary disease; CTEPH, chronic thromboembolic pulmonary hypertension; ERS, European Respiratory Society; ESC, European Society of Cardiology; PE, pulmonary embolism; PH, pulmonary hypertension; RCT, randomized controlled trial; SEC, Spanish Society of Cardiology.

**Figure 1:**
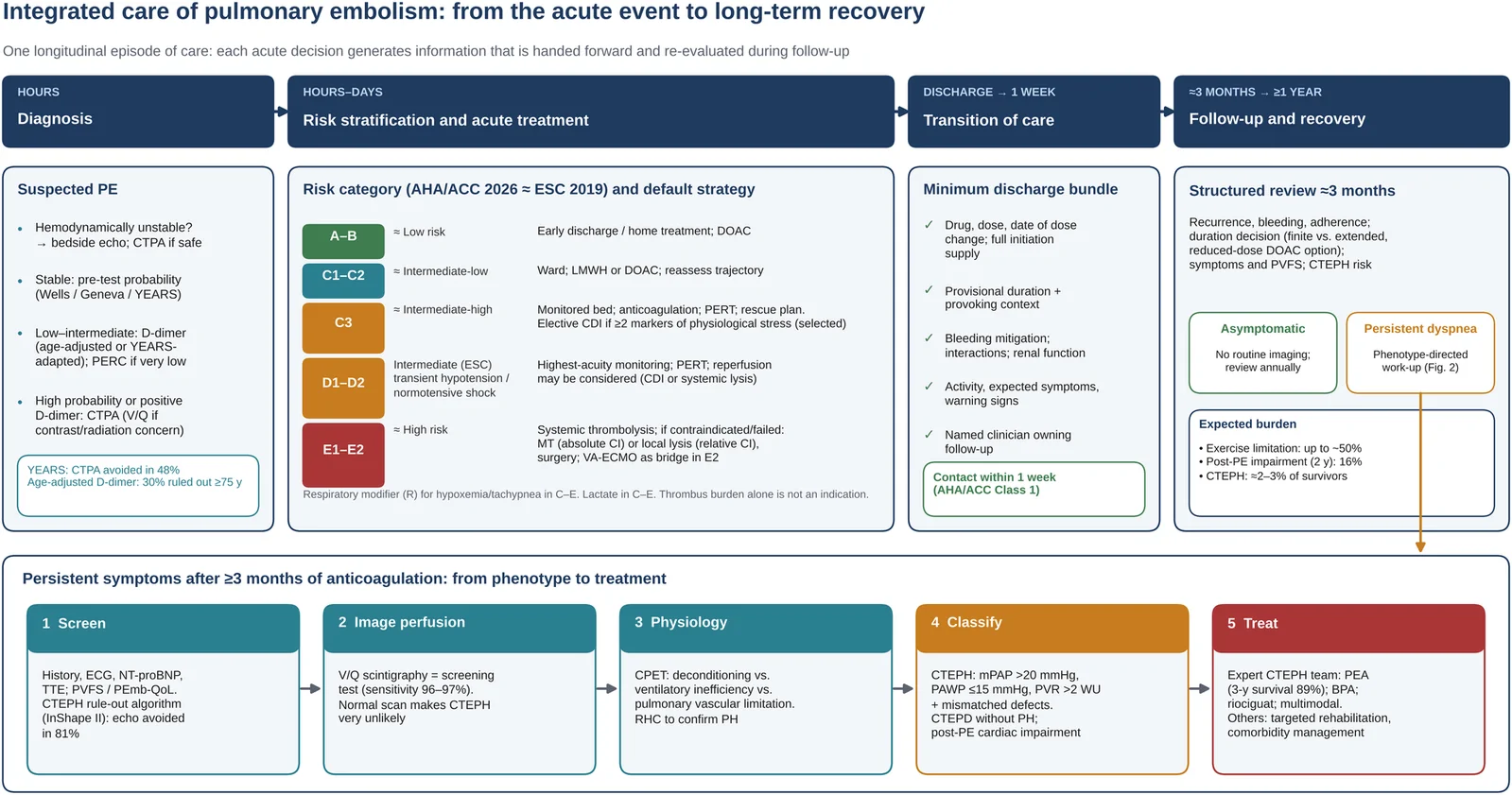
Central illustration: integrated acute-to-chronic pathway for pulmonary embolism. The upper panels follow the patient from diagnosis through risk stratification, acute treatment and transition of care to the structured three-month review; the 2026 AHA/ACC clinical categories are shown with their approximate 2019 ESC equivalents and the default strategy for each; categories C3, D1 and D2 all correspond to ESC intermediate-high risk, with D1–D2 representing its most severe end. The lower panel summarizes the phenotype-directed evaluation and treatment of persistent symptoms. AHA/ACC, American Heart Association/American College of Cardiology; BPA, balloon pulmonary angioplasty; CDI, catheter-directed intervention; CI, contraindication; CPET, cardiopulmonary exercise testing; CTEPD, chronic thromboembolic pulmonary disease; CTEPH, chronic thromboembolic pulmonary hypertension; CTPA, computed tomography pulmonary angiography; DOAC, direct oral anticoagulant; ECG, electrocardiography; ESC, European Society of Cardiology; LMWH, low-molecular-weight heparin; mPAP, mean pulmonary arterial pressure; MT, mechanical thrombectomy; NT-proBNP, N-terminal pro-B-type natriuretic peptide; PAWP, pulmonary arterial wedge pressure; PE, pulmonary embolism; PEA, pulmonary endarterectomy; PEmb-QoL, Pulmonary Embolism Quality of Life questionnaire; PERC, pulmonary embolism rule-out criteria; PERT, pulmonary embolism response team; PH, pulmonary hypertension; PVFS, Post-VTE functional status scale; PVR, pulmonary vascular resistance; RHC, right-heart catheterization; TTE, transthoracic echocardiography; VA-ECMO, venoarterial extracorporeal membrane oxygenation; V/Q, ventilation/perfusion; WU, Wood units; y, years.

## 2 Materials and methods

PubMed/MEDLINE was searched for English-language publications in adults from 1 January 2000 to 31 August 2026, complemented by the websites of major cardiovascular, hematology, emergency medicine and respiratory societies for guidelines and consensus documents, and by ClinicalTrials.gov for ongoing randomized trials. The search combined “pulmonary embolism” or “venous thromboembolism” with terms for each domain of the review (diagnosis, risk stratification, anticoagulation, thrombolysis, catheter-directed intervention, mechanical thrombectomy, embolectomy, extracorporeal support, response teams, follow-up, post-pulmonary embolism syndrome, chronic thromboembolic pulmonary disease and hypertension, balloon pulmonary angioplasty and rehabilitation); the full search strings are provided in Supplementary Table S1. Because generic terms failed to retrieve some recent trials in the initial search, the search was repeated during revision and supplemented by searches for the names of individual trials and registries (Supplementary Table S1), forward citation tracking of the 2026 AHA/ACC guideline and the 2022 ESC follow-up position paper, and screening of the reference lists of key publications.

Eligible sources were, in descending order of priority, clinical practice guidelines and society consensus documents, randomized controlled trials and their meta-analyses, prospective cohort and management studies, and large registries; smaller observational studies were included only when higher-level evidence was lacking. Case reports, conference abstracts and unpublished data were excluded. Because the review was intended to integrate clinical practice rather than to estimate pooled effects, appraisal was qualitative and no meta-analysis or formal risk-of-bias assessment was performed. To make the evidence hierarchy explicit, the type of evidence supporting each key recommendation is stated in the text and summarized in Section 11, and statements for which no supporting evidence was identified are labeled as the authors' opinion.

## 3 Pathophysiology across the acute-to-chronic continuum

### 3.1 Thrombus formation and provoking context

Most pulmonary emboli originate in the deep veins of the lower limbs or pelvis, reflecting a variable combination of venous stasis, endothelial perturbation and hypercoagulability (1). A major operation creates a strong exposure that disappears, whereas cancer, antiphospholipid syndrome, chronic inflammatory disease or persistent immobility continue to generate risk; acute infection and critical illness act mainly as transient provoking factors (14). Because the provoking context determines the risk of recurrence once anticoagulation stops, it should be recorded precisely rather than reduced to the labels “provoked” and “unprovoked” (14, 15).

### 3.2 Right ventricular failure and normotensive shock

Clinical severity is determined by the interaction between embolic obstruction and the capacity of the RV to adapt, rather than by the anatomical extent of obstruction alone (1, 9). Acute pressure overload produces RV dilatation, increased wall stress and oxygen demand, septal displacement with reduced left ventricular filling and, through systemic hypotension, a lower right coronary perfusion gradient, initiating a self-reinforcing cycle of RV ischemia and failure (1). This explains why patients with limited reserve may decompensate after a smaller embolus, and why the AHA/ACC guideline advises against using thrombus burden for risk stratification (9). Compensatory vasoconstriction can maintain systolic pressure despite falling cardiac output, producing normotensive shock with rising lactate, oliguria or altered mentation; an elevated plasma lactate in normotensive patients is independently associated with adverse short-term outcomes (16). Hypoxemia results from ventilation/perfusion mismatch, low mixed venous oxygen saturation and, occasionally, right-to-left shunting through a patent foramen ovale, so oxygen saturation is not a stand-alone measure of embolic severity (1).

### 3.3 From acute embolus to chronic thromboembolic disease

In most patients, thrombus resolves substantially during the first months of anticoagulation. In a minority, organized thrombus becomes incorporated into the arterial wall as webs, bands, stenoses, pouch lesions and occlusions; flow is redistributed and a secondary small-vessel arteriopathy can develop in unobstructed territories, increasing pulmonary vascular resistance (PVR) (8, 17). The RV initially adapts through hypertrophy and later decompensates as reserve is exhausted. Because symptoms evolve gradually, chronic disease is frequently recognized late, which is the rationale for structured follow-up after acute PE (4, 8).

## 4 Diagnosis

### 4.1 Pre-test probability and D-dimer

Diagnostic testing should begin with explicit estimation of clinical probability (1, 9). In patients with low or intermediate pre-test probability, a negative highly sensitive D-dimer safely excludes PE. An age-adjusted threshold (age   ×  10 µg/L above 50 years) increased the proportion of patients aged ≥75 years in whom PE could be excluded from 6.4% to 29.7%, with a three-month failure rate of 0.3% (18). The YEARS strategy, which combines three clinical items with probability-adapted D-dimer thresholds, avoided computed tomography pulmonary angiography (CTPA) in 48% of patients, compared with 34% under the conventional algorithm, with a three-month VTE rate of 0.61% among patients in whom PE was excluded without imaging (19). The pulmonary embolism rule-out criteria (PERC) are appropriate only in patients with very low clinical probability; in this population the PROPER cluster-randomized trial showed non-inferiority, with a three-month VTE rate of 0.1% (20). A pregnancy-adapted YEARS algorithm avoided CTPA in 39% of pregnant women with a three-month VTE rate of 0.21% (21). A positive D-dimer is not diagnostic and is particularly nonspecific in hospitalized patients, cancer, pregnancy, infection and advanced age; infection and critical illness also elevate troponin and natriuretic peptides, which should be considered when these biomarkers are used for risk stratification (1).

### 4.2 Imaging strategy

CTPA is the reference imaging test for most adults because it confirms emboli, depicts RV enlargement and may identify alternative diagnoses (1, 9). Ventilation/perfusion (V/Q) scintigraphy remains important when iodinated contrast is contraindicated, when radiation to breast tissue is a major concern (for example, in young women and during pregnancy), and when chronic thromboembolic obstruction is suspected (1, 22). Compression ultrasonography can establish VTE when proximal deep-vein thrombosis (DVT) is demonstrated, but a negative examination does not exclude PE. Bedside echocardiography is not a rule-out test in stable patients; in shock, however, acute RV pressure overload can justify immediate reperfusion when transport to definitive imaging is unsafe (1). The principal diagnostic tools are summarized in Table 2. CTPA also provides early clues to pre-existing chronic disease—eccentric wall-adherent defects, webs, bands, mosaic perfusion or enlarged bronchial arteries—which should be reported because they change the follow-up plan (8, 10).

**Table 2:** Diagnostic tools and their best clinical use.

Tool	Best use	Key performance data	Important limitation
Clinical probability score (Wells, Geneva) or YEARS items	Select the rule-out pathway	YEARS avoided CTPA in 48% of patients; 3-month VTE 0.61% when PE excluded without imaging (19)	Cannot confirm PE
High-sensitivity D-dimer (age-adjusted or probability-adapted)	Exclude PE in non-high probability	Age-adjusted cut-off: exclusion in ≥75 years increased from 6.4% to 29.7%; failure rate 0.3% (18)	Low specificity in hospitalization, cancer, pregnancy, infection and older age
PERC	Very-low-probability patients	VTE 0.1% at 3 months in PROPER (20)	Unsafe outside very-low-probability settings
CTPA	Anatomical confirmation; RV size; alternative diagnoses	Reference test in most adults (1)	Contrast, radiation, overdiagnosis of small distal defects
V/Q scintigraphy	Contrast avoidance, pregnancy, screening for CTEPD/CTEPH	Sensitivity for CTEPH 96%–97% (87)	Non-diagnostic results with abnormal chest radiograph
Compression ultrasonography	Confirm proximal DVT	Proximal DVT confirms VTE (1)	Negative result does not exclude PE
Transthoracic echocardiography	Shock assessment; RV phenotype	Allows reperfusion decisions in unstable patients (1)	Insufficient sensitivity to exclude PE

CTEPD, chronic thromboembolic pulmonary disease; CTEPH, chronic thromboembolic pulmonary hypertension; CTPA, computed tomography pulmonary angiography; DVT, deep-vein thrombosis; PE, pulmonary embolism; PERC, pulmonary embolism rule-out criteria; RV, right ventricular; VTE, venous thromboembolism; V/Q, ventilation/perfusion.

### 4.3 Incidental and isolated subsegmental pulmonary embolism

The AHA/ACC guideline assigns asymptomatic, incidentally detected PE to a separate category A (9). Incidental PE detected during cancer staging carries a meaningful recurrence risk and is treated like symptomatic PE when segmental or more proximal (23, 24). Isolated subsegmental PE is more difficult, because small defects are susceptible to artifact while treatment exposes patients with a low absolute recurrence risk to bleeding. In a prospective management study of 266 patients with subsegmental PE and no proximal DVT managed without anticoagulation, the 90-day rate of recurrent VTE was 3.1%, higher than anticipated, with a higher rate among patients with multiple subsegmental defects (25). On this evidence, surveillance without anticoagulation is defensible only for a single subsegmental defect confirmed by expert review, with negative bilateral proximal ultrasonography, adequate cardiopulmonary reserve, no active cancer or other persistent risk factor, and assured follow-up; multiple defects, reduced reserve, ongoing risk factors or inability to provide surveillance favor anticoagulation (1, 3, 25, 26).

## 5 Risk stratification: European and American frameworks

### 5.1 The 2019 ESC model

The ESC model first identifies high-risk PE, defined by hemodynamic instability: cardiac arrest, obstructive shock [systolic blood pressure (SBP) < 90 mmHg or vasopressor requirement with end-organ hypoperfusion], or persistent hypotension (SBP <90 mmHg or a drop ≥40 mmHg for >15 min not explained by other causes) (1). Normotensive patients are then classified using the Pulmonary Embolism Severity Index (PESI) (27) or its simplified version (sPESI), RV dysfunction on echocardiography or CTPA, and cardiac troponin. Intermediate-high risk requires both RV dysfunction and an elevated troponin; intermediate-low risk applies when one or neither is present in a patient with PESI class III–V or sPESI ≥1, or when either is present despite a low clinical score; low risk requires a low clinical score without RV dysfunction or troponin elevation when these are assessed (1). Quantitatively, sPESI 0 identified a 30-day mortality of 1.0% versus 10.9% for sPESI ≥1 (28), and elevated troponin was associated with a more than five-fold increase in short-term mortality (odds ratio 5.24) (29). The Hestia criteria address a different question, namely whether home treatment is feasible (Section 7.1) (30).

### 5.2 The 2026 AHA/ACC clinical categories

The 2026 AHA/ACC guideline abandoned the terms massive and submassive and introduced five clinical categories with subcategories (Table 3) (9): A, subclinical (incidental, asymptomatic) PE; B, symptomatic PE with a low severity score (sPESI 0, PESI ≤85 or Bova ≤4); C, symptomatic PE with an elevated score, subdivided by RV imaging and biomarkers (troponin, natriuretic peptide or lactate) into C1 (both normal), C2 (abnormal RV or biomarker) and C3 (abnormal RV and biomarker); D, incipient cardiopulmonary failure, comprising D1 (transient, fluid-responsive hypotension <15 min) and D2 (normotensive shock, for example lactate >2 mmol/L, oliguria or altered mentation); and E, cardiopulmonary failure, comprising E1 (persistent hypotension with cardiogenic shock) and E2 (refractory shock or cardiac arrest). A respiratory modifier (“R”) is added to categories C–E when hypoxemia, tachypnea or an escalating oxygen requirement is present (9).

**Table 3:** Side-by-side comparison of the 2019 ESC and 2026 AHA/ACC risk frameworks and key reperfusion recommendations.

2026 AHA/ACC category: definition	Approximate 2019 ESC equivalent	Systemic thrombolysis (ESC/AHA/ACC)	CDI and surgery (ESC/AHA/ACC)	Setting and other key points
A: subclinical (incidental, asymptomatic)	Not specifically classified (usually low risk)	Not indicated/not indicated	Not indicated	Treat proximal incidental PE as symptomatic PE
B: symptomatic, low severity score (sPESI 0, PESI ≤85 or Bova ≤4)	Low risk	Class III/Class 3: Harm	Not recommended	Outpatient or early discharge if criteria met (ESC IIa; AHA/ACC)
C1: elevated severity score, normal RV and biomarkers	Intermediate-low risk	Class III/Class 3: Harm	Not recommended	Ward; anticoagulation (LMWH or DOAC)
C2: elevated score, abnormal RV or ≥1 abnormal biomarker	Intermediate-low risk	Class III/Class 3: Harm	ESC: not routinely/AHA/ACC: benefit unclear (2b)	PERT evaluation (AHA/ACC Class 1 for C–E); lactate (Class 1)
C3: elevated score, abnormal RV and ≥1 abnormal biomarker	Intermediate-high risk	Routine use Class III; rescue if deterioration Class I/uncertain (2b)	ESC: alternative to rescue lysis (IIa)/AHA/ACC: benefit unclear (2b)	Monitored setting; predefined rescue plan
D1: transient hypotension (<15 min, fluid-responsive)	Intermediate-high risk (ESC requires hypotension >15 min for high risk)	Rescue if deterioration (I)/may be considered (2b)	ESC: as rescue (IIa)/AHA/ACC: may be considered (2b)	Highest-acuity monitored setting; immediate access to reperfusion
D2: normotensive shock (e.g., lactate >2 mmol/L, oliguria, altered mentation)	Intermediate-high risk (ESC requires SBP <90 mmHg for high risk)	Rescue (I)/may be considered (2b)	ESC: as rescue (IIa)/AHA/ACC: may be considered (2b)	Represents the main point of divergence between frameworks
E1: persistent or recurrent hypotension with cardiogenic shock	High risk	Class I/Class 2a	ESC: surgery (I) or CDI (IIa) if lysis contraindicated or failed/AHA/ACC: CDL, MT, surgery 2a	ICU; PERT; supportive care
E2: refractory shock or cardiac arrest	High risk	Class I/Class 2a	ESC: ECMO with surgery or CDI (IIb)/AHA/ACC: VA-ECMO 2a; surgery not preferred without circulatory support	Resuscitation area; ECMO-capable center

A respiratory modifier (R) is added to AHA/ACC categories C–E when hypoxemia, tachypnea or an escalating oxygen requirement is present. Correspondence between frameworks is approximate; categories C3, D1 and D2 all correspond to ESC intermediate-high risk, with D1 and D2 representing a more severe stage that does not meet the ESC definition of high risk (cardiac arrest, obstructive shock or persistent hypotension). Classes of recommendation are taken from the 2019 ESC guidelines (1) and the 2026 AHA/ACC guideline (9). AHA/ACC, American Heart Association/American College of Cardiology; CDI, catheter-directed intervention; CDL, catheter-directed lysis; DOAC, direct oral anticoagulant; ECMO, extracorporeal membrane oxygenation; ESC, European Society of Cardiology; ICU, intensive care unit; LMWH, low-molecular-weight heparin; MT, mechanical thrombectomy; PE, pulmonary embolism; PERT, pulmonary embolism response team; PESI, Pulmonary Embolism Severity Index; RV, right ventricular; SBP, systolic blood pressure; sPESI, simplified PESI; VA-ECMO, venoarterial ECMO.

### 5.3 Side-by-side comparison

The correspondence between the frameworks (Table 3) is approximate: categories A and B broadly correspond to ESC low-risk PE; C1 and C2 to intermediate-low risk; and C3, D1 and D2 to intermediate-high risk (1, 9). The most important difference lies at the boundary between intermediate- and high-risk disease: patients with transient hypotension (D1) or normotensive shock (D2) represent a more severe stage than C3, but they remain within the ESC intermediate-high-risk class, because the ESC model reserves high risk for cardiac arrest, obstructive shock or persistent hypotension, whereas the AHA/ACC framework recognizes them as a distinct, threatened group in which reperfusion “may be considered” (9). The AHA/ACC framework also makes lactate measurement (Class 1 in categories C–E), the respiratory modifier and incidental PE explicit, and recommends multidisciplinary PE response team (PERT) evaluation for categories C–E (9). Conversely, the principles are convergent: hemodynamic status defines urgency, RV dysfunction and myocardial injury refine prognosis in normotensive patients, routine systemic thrombolysis is not recommended in normotensive patients without signs of deterioration, and outpatient care is appropriate for selected low-risk patients (1, 9). Contributions and limitations of individual prognostic variables are summarized in Table 4.

**Table 4:** Interpretation of prognostic information in normotensive patients.

Information	Contribution	Supporting data	What it cannot decide alone
PESI or sPESI	Baseline short-term mortality estimate; identifies low-risk candidates	30-day mortality 1.0% with sPESI 0 vs. 10.9% with sPESI ≥1 (27, 28)	Need for reperfusion (driven by age and comorbidity)
Hestia criteria	Feasibility and safety of outpatient treatment	Similar 30-day adverse outcomes to sPESI triage in HOME-PE (3.8% vs. 3.6%) (30, 69)	Biological severity
Troponin	Myocardial injury	OR 5.24 for short-term mortality (29)	Cause of injury; treatment selection
BNP or NT-proBNP	RV myocardial stretch; high negative predictive value	Used as biomarker in AHA/ACC category C (1, 9)	Acute versus chronic strain
Lactate	Systemic hypoperfusion; identifies normotensive shock (AHA/ACC D2)	Independently associated with adverse outcome in normotensive PE (16)	PE-specific mechanism
RV on TTE or CTPA	RV pressure-load phenotype	Component of ESC intermediate-high risk and AHA/ACC C2–C3 (1, 9)	Net benefit of an invasive procedure
Clinical trajectory (heart rate, SBP, respiratory rate, oxygen requirement)	Early warning of deterioration	≥2 markers of physiological stress defined the HI-PEITHO population (31)	Cause of deterioration (exclude bleeding, sepsis, arrhythmia)

AHA/ACC, American Heart Association/American College of Cardiology; BNP, B-type natriuretic peptide; CTPA, computed tomography pulmonary angiography; ESC, European Society of Cardiology; NT-proBNP, N-terminal pro-B-type natriuretic peptide; OR, odds ratio; PE, pulmonary embolism; PESI, Pulmonary Embolism Severity Index; RV, right ventricular; SBP, systolic blood pressure; sPESI, simplified Pulmonary Embolism Severity Index; TTE, transthoracic echocardiography.

### 5.4 Clinical implications of categories C, D and E

Category C comprises hospitalized patients who rarely benefit from therapies beyond anticoagulation; its subdivision by RV imaging and biomarkers, with lactate measurement (Class 1), is intended to identify those at risk of progression (9). Category C3, particularly with a respiratory modifier, resembles the PEITHO and HI-PEITHO populations, in whom the role of CDI is debated (Section 6.5) (31, 32).

Category D is the main conceptual advance of the 2026 framework. It recognizes incipient cardiopulmonary failure in patients whose blood pressure is not persistently low: transient, fluid-responsive hypotension (D1) and normotensive shock (D2), defined by markers of hypoperfusion such as lactate >2 mmol/L, acute kidney injury, oliguria, altered mentation or a cardiac index <2.2 L/min/m^2^ (9). In the FLASH registry, approximately one-third of normotensive patients with intermediate-risk PE who underwent invasive hemodynamic assessment had a cardiac index in the shock range (33), and a composite shock score combining clinical, biochemical and imaging variables has been proposed to identify them non-invasively (34). Clinically, category D implies admission to an intensive care or high-dependency unit, immediate PERT involvement, avoidance of sedation, intubation and aggressive fluid loading, early vasopressor or inotropic support, and readiness for reperfusion, for which the AHA/ACC guideline assigns a Class 2b recommendation (9). The evidence base is, however, limited: the concept derives largely from retrospective analyses of patients undergoing mechanical thrombectomy, and no prospective study has yet shown that treating normotensive shock differently improves outcomes (9, 33).

Category E corresponds to ESC high-risk PE and mandates immediate reperfusion and circulatory support; by distinguishing refractory shock or arrest (E2), the framework specifies when VA-ECMO is reasonable and surgery without circulatory support is not preferred (Sections 6.3 and 6.6) (1, 9).

### 5.5 Translating categories into monitoring and escalation

A single baseline label is insufficient because patients may progress from stable RV strain to incipient failure, a trajectory formalized by the AHA/ACC D categories (9). In the authors' opinion, the admission note of every patient at ESC intermediate-high risk or in AHA/ACC category C3–D should document explicit escalation triggers (new hypotension, rising lactate, increasing oxygen requirement, syncope, altered mentation, oliguria, progressive tachycardia or worsening RV failure), the pre-agreed rescue strategy and the responsible team, consistent with the recommendation for PERT involvement in these categories (1, 9).

## 6 Acute treatment

### 6.1 Initial stabilization

Supportive therapy should protect RV perfusion and avoid iatrogenic collapse. Because aggressive volume loading may worsen RV dilatation and left ventricular underfilling, cautious fluid administration (≤500 mL) is reserved for patients with low central venous pressure, and norepinephrine is the first-line vasopressor for hypotension (1). Induction of anesthesia and positive-pressure ventilation can precipitate collapse by reducing venous return and increasing RV afterload; the AHA/ACC guideline therefore advises against deep sedation and intubation unless clinically necessary in patients with RV dysfunction (1, 9). Venoarterial extracorporeal membrane oxygenation (VA-ECMO) may stabilize refractory shock or cardiac arrest as a bridge to reperfusion or recovery, but it is not definitive thrombus therapy (Section 6.6) (9, 35).

### 6.2 Anticoagulation

Anticoagulation should begin promptly, including while awaiting imaging when clinical probability is high or intermediate (1). Low-molecular-weight heparin (LMWH) is preferred over unfractionated heparin (UFH) for parenteral therapy in most patients because of more predictable pharmacokinetics and lower rates of recurrence without increased bleeding (1, 9); UFH remains preferable when immediate reperfusion or another procedure is anticipated, when rapid reversal may be needed, and in severe renal impairment (1). For most eligible adults, direct oral anticoagulants (DOACs) are preferred over vitamin K antagonists (VKAs). In a meta-analysis of the phase 3 trials, DOACs had similar efficacy for recurrent VTE [relative risk (RR) 0.90] and reduced major bleeding (RR 0.61), intracranial hemorrhage (RR 0.37) and fatal bleeding (36–40). Apixaban and rivaroxaban can be started without a parenteral lead-in, whereas dabigatran and edoxaban require at least five days of parenteral anticoagulation. In the randomized COBRRA trial, clinically relevant bleeding during three months of treatment was lower with apixaban than with rivaroxaban (3.3% vs. 7.1%; RR 0.46) (41).

Drug choice must account for renal and hepatic function, pregnancy, antiphospholipid syndrome, body weight, interactions, adherence, affordability, cancer site, thrombocytopenia and planned procedures (Table 5). In cancer-associated VTE, factor Xa inhibitors were non-inferior or superior to dalteparin for recurrence, but edoxaban and rivaroxaban increased gastrointestinal and genitourinary bleeding in patients with luminal tumors, whereas apixaban did not increase major bleeding (3.8% vs. 4.0%) (24, 42–44). In high-risk antiphospholipid syndrome, rivaroxaban caused an excess of thrombotic and bleeding events compared with warfarin, and VKAs remain the agents of choice (45); recommendations for obesity and pregnancy are summarized in Table 5 (22, 46).

**Table 5:** Anticoagulant selection across common clinical contexts.

Context	Preferred approach	Key evidence	Key caution
Most stable adults	Apixaban or rivaroxaban (single-drug approach); dabigatran or edoxaban after ≥5 days of parenteral anticoagulation	DOAC vs. VKA: major bleeding RR 0.61, intracranial hemorrhage RR 0.37 (36); apixaban vs. rivaroxaban: clinically relevant bleeding 3.3% vs. 7.1% (41)	Renal function, interactions, adherence, dose transition
Hemodynamic instability or procedure likely	Intravenous UFH	Allows rapid adjustment and reversal (1)	Monitoring variability; heparin-induced thrombocytopenia
Pregnancy	Therapeutic LMWH	Recommended by ASH and ESC (1, 22)	Weight-based dosing; written peripartum plan; DOAC and VKA avoided
Active cancer	Apixaban, edoxaban, rivaroxaban or LMWH	Recurrence reduced vs. dalteparin; more GI/GU bleeding with edoxaban and rivaroxaban (42–44)	Luminal GI/GU lesions, thrombocytopenia, interactions
High-risk (triple-positive) antiphospholipid syndrome	VKA	Excess events with rivaroxaban in TRAPS (45)	Avoid DOACs
Obesity (BMI >40 kg/m^2^ or weight >120 kg)	Standard-dose apixaban or rivaroxaban, or VKA	ISTH guidance (46)	Bariatric surgery: absorption uncertain
Severe renal impairment	UFH acutely; individualized VKA-based strategy	Renally cleared agents accumulate (1)	DOACs and LMWH require dose review or avoidance

ASH, American Society of Hematology; BMI, body mass index; DOAC, direct oral anticoagulant; ESC, European Society of Cardiology; GI, gastrointestinal; GU, genitourinary; ISTH, International Society on Thrombosis and Haemostasis; LMWH, low-molecular-weight heparin; RR, relative risk; UFH, unfractionated heparin; VKA, vitamin K antagonist.

### 6.3 Systemic thrombolysis

Systemic thrombolysis is recommended for high-risk PE (ESC Class I, level of evidence B; AHA/ACC category E, Class 2a) when bleeding risk is acceptable (1, 9). In a meta-analysis of 16 trials, thrombolysis was associated with lower all-cause mortality (odds ratio 0.53) but higher major bleeding (9.24% vs. 3.42%) and intracranial hemorrhage (1.46% vs. 0.19%) (47). In intermediate-high-risk PE, the PEITHO trial showed that full-dose tenecteplase reduced death or hemodynamic decompensation within 7 days (2.6% vs. 5.6%; odds ratio 0.44), but increased major extracranial bleeding (6.3% vs. 1.2%) and hemorrhagic stroke (2.0% vs. 0.2%), without a mortality advantage (32). Its long-term follow-up showed no benefit on survival, functional limitation or CTEPH (Section 9.2) (48). Accordingly, both guidelines recommend against routine systemic thrombolysis in normotensive patients (ESC Class III; AHA/ACC categories A–C2, Class 3: Harm) and favor anticoagulation with close monitoring and rescue reperfusion if deterioration occurs (ESC Class I for rescue thrombolysis) (1, 9). For categories D1–D2, systemic thrombolysis “may be considered” (Class 2b) (9).

Reduced-dose thrombolysis is attractive: 50 mg of alteplase was as effective as 100 mg on radiological endpoints with fewer bleeding events (49), but such trials have been underpowered for clinical outcomes. The ongoing PEITHO-3 trial is randomizing approximately 650 patients with intermediate-high-risk PE and additional clinical markers of severity to reduced-dose alteplase or placebo on top of heparin, with a 30-day composite of death, hemodynamic decompensation or recurrent PE as the primary outcome (50). Contraindications should be weighed against the threat of imminent death from obstructive shock (1).

### 6.4 Catheter-directed intervention: terminology and evidence

For clarity, we use catheter-directed intervention (CDI) as an umbrella term for percutaneous reperfusion techniques, comprising two families (11, 12). Local (catheter-directed) thrombolysis delivers a low total dose of fibrinolytic—typically 8–24 mg of alteplase over several hours—through standard multi-side-hole catheters (conventional catheter-directed thrombolysis, CDT) or ultrasound-facilitated catheters (USCDT). Mechanical thrombectomy (MT) removes thrombus without, or with minimal, fibrinolytic exposure, using large-bore aspiration or extraction systems or computer-assisted vacuum thrombectomy. The modalities are compared in Table 6 and the principal trials in Table 7.

**Table 6:** Comparison of reperfusion and support strategies.

Strategy	Mechanism	Potential advantage	Principal limitation	Typical role
Systemic thrombolysis	Full-dose intravenous fibrinolytic	Rapid; widely available; mortality benefit in high-risk PE (47)	Major bleeding 9.2%; intracranial hemorrhage 1.5% (47)	High-risk (category E) PE without contraindication
CDI – local thrombolysis (standard catheter or USCDT)	Low-dose fibrinolytic (typically 8–24 mg alteplase) over several hours	Lower fibrinolytic exposure; reduced early decompensation in HI-PEITHO (31)	Still exposes to a fibrinolytic; infusion time; access-site and major bleeding (≈7%–10% in PEERLESS and SEATTLE II); rare pulmonary artery injury (54, 60, 62)	Intermediate-high risk with physiological stress (selected); high risk with relative contraindication to systemic lysis
CDI – mechanical thrombectomy (large-bore aspiration or computer-assisted vacuum)	Immediate thrombus removal without fibrinolytic	No lytic exposure; immediate hemodynamic effect; less ICU use than CDL in PEERLESS (60)	Large-bore access with access-site complications (55, 60, 62); blood loss, now mitigated by blood-return systems (e.g., FlowSaver) (12); rare pulmonary artery perforation or right-heart injury (62); no mortality data from RCTs	High risk with absolute contraindication to or failure of lysis; selected intermediate-high risk
Surgical embolectomy	Open thrombus removal	Definitive central clot removal; treats thrombus in transit	Requires expert surgical program; in-hospital mortality 27% in nationwide data (63)	Contraindicated or failed lysis; thrombus straddling a PFO
VA-ECMO	Circulatory and respiratory support	Immediate support in refractory shock or arrest	High complication burden; not definitive therapy (35)	Bridge to MT, surgery or recovery (category E2)

CDI, catheter-directed intervention; CDL, catheter-directed lysis; ECMO, extracorporeal membrane oxygenation; ICU, intensive care unit; MT, mechanical thrombectomy; PE, pulmonary embolism; PFO, patent foramen ovale; RCT, randomized controlled trial; USCDT, ultrasound-facilitated catheter-directed thrombolysis; VA-ECMO, venoarterial ECMO.

**Table 7:** Key randomized trials and prospective studies of catheter-directed intervention.

Study (design, n)	Population	Comparison	Primary outcome and main result	Safety
ULTIMA (RCT, 59) (51)	Intermediate risk	USCDT + heparin vs. heparin	RV/LV reduction at 24 h: 0.30 vs. 0.03	No major bleeding
OPTALYSE (RCT, 101) (52)	Intermediate risk	USCDT, 4 regimens (8–24 mg alteplase, 2–6 h)	RV/LV improved in all arms	4 major bleeds, 1 ICH
SUNSET sPE (RCT, 81) (53)	Intermediate risk	USCDT vs. standard CDT	No difference in thrombus burden reduction	Similar bleeding
CANARY (RCT, 94) (59)	Intermediate-high risk	Conventional CDT vs. anticoagulation	RV/LV >0.9 at 3 months: 4.3% vs. 12.8% (NS); stopped early	BARC 3 or 5 bleeding 2.1% vs. 0%
FLASH (registry, 800) (55)	Intermediate and high risk	Large-bore MT (single arm)	MAE 1.8%; 30-day all-cause mortality 0.8%	Low major bleeding
FLAME (prospective, non-randomized, 115) (58)	High risk	Large-bore MT vs. context arm (mostly systemic lysis)	In-hospital composite 17.0% (performance goal 32.0%); mortality 1.9% vs. 29.5%	Groups not comparable
HI-PEITHO (RCT, 544) (31)	Intermediate-high risk + ≥ 2 markers of physiological stress	USCDT + anticoagulation vs. anticoagulation	7-day PE death, decompensation or recurrence: 4.0% vs. 10.3% (RR 0.39, 95% CI 0.20–0.77); driven by decompensation	No ICH; 7-day major bleeding 4.1% vs. 2.2% (NS)
PEERLESS (RCT, 550) (60)	Intermediate risk with additional risk features	Large-bore MT vs. CDT	Win ratio 5.01 (95% CI 3.68–6.97); deterioration/bailout 1.8% vs. 5.4%; ICU use 41.6% vs. 98.6%	No difference in mortality, ICH or major bleeding
STORM-PE (RCT, 100) (61)	Intermediate-high risk	Computer-assisted vacuum thrombectomy + anticoagulation vs. anticoagulation	RV/LV reduction at 48 h: 0.52 vs. 0.24 (difference 0.27)	7-day MAE 4.3% vs. 7.5%

BARC, Bleeding Academic Research Consortium; CDT, catheter-directed thrombolysis; CI, confidence interval; ICH, intracranial hemorrhage; ICU, intensive care unit; MAE, major adverse events; MT, mechanical thrombectomy; NS, not significant; PE, pulmonary embolism; RCT, randomized controlled trial; RR, relative risk; RV/LV, right ventricular-to-left ventricular diameter ratio; USCDT, ultrasound-facilitated catheter-directed thrombolysis.

Early evidence consisted of single-arm studies or small randomized trials with surrogate endpoints, chiefly the reduction in the RV-to-left ventricular (RV/LV) diameter ratio at 24–48 h: ULTIMA, SEATTLE II, OPTALYSE and SUNSET sPE for local thrombolysis (51–54), and FLARE, EXTRACT-PE and the 800-patient FLASH registry for MT (55–57). In high-risk PE, the non-randomized FLAME study reported in-hospital mortality of 1.9% with large-bore MT versus 29.5% in a context arm treated predominantly with systemic thrombolysis, although the groups were not comparable, so these data represent observational evidence only (58). The small CANARY trial was stopped early without a significant difference in its primary endpoint (59) (Table 7).

The evidence base has changed substantially with four randomized trials (Table 7). HI-PEITHO randomized 544 patients with intermediate-high-risk PE (RV/LV ≥1.0, elevated troponin) and at least two markers of physiological stress (heart rate ≥100/min, SBP ≤110 mmHg, respiratory rate ≥20/min or oxygen saturation ≤90%) to USCDT plus anticoagulation or anticoagulation alone (31). The 7-day composite of PE-related death, cardiorespiratory decompensation or collapse, or symptomatic recurrence occurred in 4.0% versus 10.3% (RR 0.39; 95% confidence interval [CI] 0.20–0.77), corresponding to a number needed to treat of approximately 16. The effect was driven by cardiorespiratory decompensation (3.7% vs. 10.3%), defined to include a National Early Warning Score ≥9; PE-related death (1.1% vs. 0.4%) and 30-day all-cause mortality (1.8% vs. 1.1%) did not differ, there was no intracranial hemorrhage, and 7-day major bleeding was numerically but not significantly higher (4.1% vs. 2.2%) (31). In PEERLESS (550 patients), large-bore MT was superior to CDT on a hierarchical win ratio (5.01; 95% CI 3.68–6.97) driven by less clinical deterioration or bailout (1.8% vs. 5.4%) and lower intensive care unit (ICU) use, without differences in mortality or bleeding (60). In STORM-PE (100 patients), computer-assisted vacuum thrombectomy plus anticoagulation reduced the 48-hour RV/LV ratio more than anticoagulation alone (0.52 vs. 0.24) (61).

HI-PEITHO is thus the first adequately powered randomized trial to show that a CDI reduces early clinical deterioration. However, it was open-label and industry-funded, enrolled highly selected patients at experienced centers, relied on a composite in which a physiological score contributed substantially, and did not show reductions in mortality; long-term functional outcomes are pending (31). PEERLESS compared two interventional strategies with a resource-driven endpoint, and STORM-PE used a surrogate endpoint (60, 61). Ongoing trials, including PEERLESS II (large-bore MT versus anticoagulation), PE-TRACT (CDI versus anticoagulation with functional endpoints) and PEITHO-3, will further define net benefit (Section 11).

### 6.5 Elective CDI in intermediate-high-risk PE with several adverse prognostic markers

Whether such patients should undergo elective CDI rather than anticoagulation with rescue therapy is the most debated question in acute PE care. Arguments in favor include the 61% relative reduction in early decompensation in HI-PEITHO (31), the low rates of intracranial hemorrhage with CDI compared with systemic thrombolysis (31, 47, 60), and the position statement of the Spanish Society of Cardiology, which suggests considering elective CDI within 24 h in intermediate-high-risk patients with multiple poor prognostic factors (for example, elevated lactate, syncope, borderline blood pressure or tachycardia) (12). The AHA/ACC guideline assigns a Class 2b recommendation (benefit unclear) to CDI in categories C2–C3 and “may be considered” in D1–D2 (9). Arguments for caution include the absence of a mortality benefit, the lack of long-term benefit after thrombolysis in PEITHO (48), procedural complications (12, 62), costs, unequal access, and the fact that most patients treated with anticoagulation alone in HI-PEITHO and PEITHO did not deteriorate (31, 32).

CDI-related complications can be grouped into access-site and vascular complications (hematoma, inadvertent arterial puncture, retroperitoneal hemorrhage, pseudoaneurysm and arteriovenous fistula); bleeding, including intracranial hemorrhage when a fibrinolytic is used; injury to the pulmonary arteries (perforation or pseudoaneurysm, presenting with hemoptysis, hemothorax or desaturation) or to the right heart (tricuspid valve damage, perforation or tamponade, and arrhythmias including atrioventricular block); periprocedural hemodynamic deterioration; and device-related problems such as catheter entrapment or kinking (62). Their reported frequency varies widely, because definitions are not standardized and reporting windows range from 48 h to 90 days (62). In the randomized trials and prospective registries summarized in a recent expert review, access-site complications occurred in approximately 1%–8% of patients, the overall rate of pulmonary artery injury was around 0.7%, and intracranial hemorrhage occurred in 0%–2% (62). Major bleeding ranged from approximately 1% with large-bore MT in the FLASH registry to approximately 7% with either MT or CDT in PEERLESS and 10% with USCDT in SEATTLE II, and it reached 11% after MT in high-risk patients in FLAME (54, 55, 58, 60). Randomized trials report no or very low procedural mortality, whereas real-world data report CDI-related mortality of 3.5%–6.7%, and in the US Food and Drug Administration Manufacturer and User Facility Device Experience (MAUDE) database, pulmonary vascular perforation was the most common complication resulting in death after mechanical thrombectomy (62). Large-bore aspiration also entails blood loss, which is now mitigated by blood-return systems (12). These data support performing CDI at experienced centers, with real-time ultrasound-guided venous access, careful catheter and guidewire manipulation and predefined bail-out options such as systemic thrombolysis or surgery, and weighing the expected benefit against procedural risk in each patient (12, 62).

Thus, the evidence supporting elective CDI consists of one randomized trial with an early clinical endpoint, surrogate-endpoint trials and registries, and expert consensus. In the authors' opinion, pending PEITHO-3, PEERLESS II and PE-TRACT, elective CDI is reasonable after PERT discussion for patients who meet HI-PEITHO-like criteria (ESC intermediate-high risk or AHA/ACC category C3 with at least two markers of physiological stress) or AHA/ACC category D1–D2, have low procedural risk and are treated at experienced centers; other normotensive patients should receive anticoagulation with monitoring and a predefined rescue strategy.

### 6.6 High-risk PE when systemic thrombolysis is contraindicated or has failed

For high-risk PE with a contraindication to, or failure of, systemic thrombolysis, the ESC recommends surgical embolectomy (Class I, level C) and suggests percutaneous catheter-directed treatment as an alternative (Class IIa, level C) (1); the AHA/ACC guideline considers local thrombolysis, MT and surgical embolectomy reasonable in category E1 (Class 2a) and VA-ECMO reasonable in category E2 with refractory shock (Class 2a) (9). No randomized trial has compared MT with local thrombolysis in this setting, so their relative roles rest on mechanistic reasoning, registry data and expert consensus rather than on comparative trials (11, 12, 58). MT provides immediate debulking without fibrinolytic exposure and is generally preferred when the contraindication is absolute (for example, recent intracranial hemorrhage or active bleeding) or when the patient cannot await a multi-hour infusion; local thrombolysis, which still exposes the patient to a low-dose fibrinolytic, may be considered when contraindications are relative, when MT is unavailable, or after incomplete mechanical debulking (11, 12). In refractory shock or cardiac arrest, VA-ECMO can serve as a bridge to MT or surgical embolectomy; observational case series suggest better outcomes when ECMO is combined with definitive reperfusion than when it is used as stand-alone therapy, but no randomized data exist (9, 35).

### 6.7 Surgical embolectomy, right-heart thrombus and PE response teams

Surgical embolectomy is an option for high-risk PE when thrombolysis is contraindicated or has failed, and for thrombus in transit or straddling a patent foramen ovale (1, 9); outcomes depend heavily on selection and experience, with an in-hospital mortality of 27.2% in a nationwide United States cohort (63). Evidence for surgical embolectomy is limited to observational series. Management of right-heart thrombus should be individualized by a multidisciplinary team according to hemodynamic status, thrombus mobility and size, bleeding risk, patent foramen ovale and local expertise, as randomized evidence is lacking (1). The AHA/ACC guideline recommends multidisciplinary PERT evaluation for categories C–E (Class 1) (9). This recommendation rests on observational before-and-after studies: in a meta-analysis, PERT implementation increased the use of advanced therapies (RR 2.67), whereas the reduction in mortality was not significant (RR 0.89; 95% CI 0.67–1.19) (64, 65). A PERT should therefore be judged by timeliness, appropriateness, complications and patient-centered outcomes, not by procedural volume (authors' opinion).

### 6.8 Inferior vena cava filters

Inferior vena cava filters do not treat PE. In PREPIC2, adding a retrievable filter to anticoagulation did not reduce recurrent PE at three months (3.0% vs. 1.5%; RR 2.00) (66). Filters should be reserved for acute VTE with an absolute contraindication to anticoagulation or recurrence despite adequate anticoagulation, with a documented retrieval plan (1, 9).

## 7 Transitions of care: disposition, discharge and early follow-up

### 7.1 Outpatient treatment and early discharge

Selected low-risk patients (ESC low risk; AHA/ACC categories A–B) can be treated at home or discharged early (1, 9). Outpatient treatment was non-inferior to inpatient care in the randomized OTPE trial (recurrent VTE 0.6% vs. 0% at 90 days) (67); early discharge on rivaroxaban was associated with recurrent VTE or PE-related death in 0.6% in HoT-PE (68); and in HOME-PE, Hestia- and sPESI-based triage yielded similar 30-day adverse outcomes (3.8% vs. 3.6%) (69). A low clinical score is necessary but not sufficient. Oxygen requirement, bleeding risk, renal function, comorbidity, pain control, medication access, health literacy and social support must also be favorable (30, 69). In the authors' opinion, when medication access, housing, transport, language support or follow-up are unreliable, a brief admission may be safer than nominally guideline-concordant discharge.

### 7.2 The minimum discharge bundle

In the authors' experience, the most preventable anticoagulant errors occur at transitions: the initial high-dose phase of apixaban and rivaroxaban, the parenteral lead-in for dabigatran and edoxaban, VKA overlap, duplication with residual heparin and prescriptions that do not cover the initiation phase. We therefore propose a minimum discharge bundle (Table 8) that specifies the drug, dose and date of any dose change, the provisional duration and provoking context, bleeding mitigation, warning signs and a named clinician responsible for follow-up. The bundle operationalizes the AHA/ACC recommendations for clinical contact within the first week after discharge (Class 1), a visit at or before three months to decide on anticoagulation duration (Class 1), and systematic enquiry about PE-related symptoms and functional limitation at every visit for at least one year (Class 1) (9), as well as the ESC recommendation for routine re-evaluation three to six months after acute PE (Class I) (1, 4).

**Table 8:** Minimum discharge bundle and longitudinal follow-up schedule after acute pulmonary embolism.

Time point/domain	Required element	Failure prevented	Basis
Discharge – anticoagulant	Drug, dose, start date, date of dose change (lead-in or step-down), missed-dose advice, prescription covering the full initiation phase	Underdosing, duplication, interruption	Authors' proposal
Discharge – duration	Provisional review date and recorded provoking context	Indefinite treatment by inertia or premature stop	(1, 14)
Discharge – safety	Bleeding signs, interacting drugs, renal and hepatic monitoring	Avoidable major bleeding	(1)
Discharge – recovery	Graded activity, expected symptoms, urgent warning signs	Fear-driven inactivity; delayed recognition of deterioration	(9)
Discharge – acute-phase handover dataset	Diagnostic certainty; AHA/ACC and ESC risk category; RV and biomarker findings (including lactate); reperfusion therapy and complications; CTPA signs of chronic thromboembolism	Loss of information needed to interpret later symptoms and CTEPH risk	(4, 84); authors' proposal
Discharge – ownership	Named clinician responsible for follow-up and duration decision	Loss to follow-up	Authors' proposal
Within 1 week	Contact: adherence, access barriers, bleeding, education	Early errors and non-adherence	AHA/ACC Class 1 (9)
≈3 months (3–6 months)	Duration decision; symptoms and functional status (PVFS); CTEPH risk assessment; echocardiography if symptomatic	Unnecessary extension or premature stop; delayed CTEPH diagnosis	ESC Class I; AHA/ACC Class 1 (1, 4, 9)
Every visit for ≥1 year	Enquire about PE-related symptoms and functional limitation; anxiety and depression	Unrecognized post-PE syndrome	AHA/ACC Class 1 and 2a (9)
Persistent symptoms	V/Q scintigraphy; referral to CTEPH center when mismatched defects persist	Delayed diagnosis of operable disease	ESC Class I (1, 10)
Extended therapy	Annual review of bleeding risk, renal function and preference	Unbalanced net clinical benefit	(1, 9)

AHA/ACC, American Heart Association/American College of Cardiology; CTEPH, chronic thromboembolic pulmonary hypertension; ESC, European Society of Cardiology; PE, pulmonary embolism; PVFS, Post-VTE Functional Status scale; V/Q, ventilation/perfusion.

Planned interruptions should be scheduled rather than improvised: heparin bridging during DOAC interruption is unnecessary and increases bleeding (70), and, in the authors' opinion, elective procedures should be deferred until the initial treatment phase has been completed whenever possible.

### 7.3 Communication and shared decision-making

Shared decision-making is essential when evidence supports more than one reasonable strategy: surveillance versus anticoagulation for isolated subsegmental PE, finite versus extended therapy, reduced-dose secondary prevention, elective CDI, and intervention for symptomatic CTEPD without resting pulmonary hypertension (4, 9). The AHA/ACC guideline considers screening for anxiety and depression reasonable (Class 2a) (9). In the authors' opinion, explaining hemodynamic risk and the expected recovery trajectory is more informative for patients than anatomical terms such as “massive”.

## 8 Duration of anticoagulation and secondary prevention

### 8.1 The duration decision

All patients require at least three months of therapeutic anticoagulation; thereafter, the decision balances the risk of recurrence if treatment stops, the risk of bleeding if it continues, and patient preference (1, 9). After a first unprovoked VTE, the cumulative risk of recurrence after stopping anticoagulation is approximately 10% at one year, 16% at two years, 25% at five years and 36% at ten years (15). A major transient provoking factor, such as major surgery, supports discontinuation after three months, whereas unprovoked PE, persistent risk factors, active cancer and recurrent VTE favor extended therapy (1, 14, 26, 71). The AHA/ACC guideline recommends continuing anticoagulation—with reduced-dose apixaban or rivaroxaban as an option—after the initial three months unless the PE was a first event strongly provoked by a transient risk factor (Class 1) (9).

Reduced-dose DOACs combine efficacy with a favorable bleeding profile. In AMPLIFY-EXT, recurrent VTE or VTE-related death occurred in 8.8% with placebo versus 1.7% with apixaban 2.5 mg twice daily, with similar major bleeding (72); in EINSTEIN CHOICE, recurrence was 1.2% with rivaroxaban 10 mg versus 4.4% with aspirin (73). In RENOVE, which enrolled patients at high risk of recurrence, reduced-dose DOACs were non-inferior to full-dose DOACs for recurrent VTE (2.2% vs. 1.8%) and reduced clinically relevant bleeding (9.9% vs. 15.2%; hazard ratio 0.61) (74). In API-CAT, reduced-dose apixaban was non-inferior to full-dose apixaban for extended treatment of cancer-associated VTE (recurrence 2.1% vs. 2.8%), with less clinically relevant bleeding (12.1% vs. 15.6%) (75).

### 8.2 Dynamic reassessment of bleeding risk

Bleeding risk is dynamic: age, kidney function, anemia, antiplatelet or nonsteroidal anti-inflammatory drug use, uncontrolled hypertension, alcohol, falls and prior bleeding should be reviewed at each visit and modified where possible (1, 4). In the authors' opinion, neither a previous PE nor a bleeding score should replace shared decision-making, and in older adults falls rarely justify withholding anticoagulation in isolation.

### 8.3 Special populations

In cancer-associated PE, thrombotic and bleeding risks vary with tumor site, mucosal involvement, anticancer therapy, thrombocytopenia and prognosis, and anticoagulation generally continues while cancer is active (24). Thrombocytopenia requires a graded strategy; full-dose anticoagulation is usually continued above 50 × 10⁹/L, whereas dose reduction, platelet support or temporary interruption are considered below this threshold depending on clot burden and time from the acute event (76). After estrogen-associated PE, estrogen-containing contraception is usually replaced by progestin-only or nonhormonal methods before anticoagulation ends; in an analysis of the EINSTEIN trials, continuing hormonal therapy during anticoagulation was not associated with an excess of recurrent VTE, whereas abnormal uterine bleeding was more frequent with rivaroxaban than with enoxaparin/VKA (77). Pregnancy-associated PE is treated with LMWH for at least six weeks postpartum and for a minimum total duration of three months, and future pregnancies require a prophylaxis plan (1, 22). In severe or rapidly changing renal dysfunction, UFH may offer controllability, and DOAC selection must reflect drug clearance (1).

### 8.4 Suspected recurrence during anticoagulation

Suspected recurrence requires objective confirmation and comparison with previous imaging, because residual abnormalities may persist (1, 4). The evaluation should establish the drug and dose, adherence, interruptions, malabsorption, renal and hepatic function and interacting medication; when recurrence is confirmed despite verified exposure, cancer, antiphospholipid syndrome and heparin-induced thrombocytopenia should be considered, and a temporary switch to LMWH is commonly used (authors' opinion).

## 9 Post-PE syndrome, CTEPD and CTEPH

### 9.1 Definitions and hemodynamic thresholds

The post-pulmonary embolism (post-PE) syndrome is an umbrella term for new or persistent dyspnea, exercise limitation or impaired quality of life after at least three months of adequate anticoagulation that is not explained by other conditions (4, 5). It comprises distinct phenotypes (Table 9): symptoms without demonstrable cardiopulmonary impairment, frequently related to deconditioning or dysfunctional breathing; post-PE cardiac impairment with persistent RV dysfunction; CTEPD without pulmonary hypertension; and CTEPH (4).

**Table 9:** Definitions and phenotype-based management after pulmonary embolism.

Phenotype	Defining pattern	Hemodynamics (RHC)	Management emphasis
Recovered	Symptoms and function near baseline	Not required	Duration decision; recurrence prevention
Symptoms without cardiopulmonary impairment	Persistent symptoms with nondiagnostic resting evaluation; deconditioning or dysfunctional breathing	Not required	CPET; targeted rehabilitation; breathing and psychological assessment (91)
Post-PE cardiac impairment	Persistent RV dysfunction without established CTEPH	If PH suspected	Serial cardiac assessment; exclude competing cardiac diagnoses (4)
CTEPD without PH	Symptoms with mismatched perfusion defects and chronic thromboembolic changes after ≥3 months of anticoagulation	mPAP ≤20 mmHg at rest; possible exercise PH (mPAP/CO slope >3 mmHg/L/min)	Expert causal assessment; selected PEA or BPA (8, 100)
CTEPH	As above, with pre-capillary PH	mPAP >20 mmHg, PAWP ≤15 mmHg, PVR >2 WU	Multidisciplinary CTEPH team: PEA, BPA, riociguat, multimodal therapy; lifelong anticoagulation (10)

Hemodynamic thresholds according to the 2022 ESC/ERS guidelines (10). BPA, balloon pulmonary angioplasty; CO, cardiac output; CPET, cardiopulmonary exercise testing; CTEPD, chronic thromboembolic pulmonary disease; CTEPH, chronic thromboembolic pulmonary hypertension; mPAP, mean pulmonary arterial pressure; PAWP, pulmonary arterial wedge pressure; PE, pulmonary embolism; PEA, pulmonary endarterectomy; PH, pulmonary hypertension; PVR, pulmonary vascular resistance; RHC, right-heart catheterization; RV, right ventricular; WU, Wood units.

According to the 2022 ESC/ERS guidelines, pulmonary hypertension is defined at right-heart catheterization by a mean pulmonary arterial pressure (mPAP) > 20 mmHg, and pre-capillary pulmonary hypertension additionally requires a pulmonary arterial wedge pressure (PAWP) ≤ 15 mmHg and a PVR >2 Wood units (WU); exercise pulmonary hypertension is defined by an mPAP/cardiac output slope >3 mmHg/L/min between rest and exercise (10). CTEPH is pre-capillary pulmonary hypertension with mismatched perfusion defects and imaging signs of chronic thromboembolic obstruction after at least three months of effective anticoagulation (8, 10). CTEPD is the broader term for symptomatic patients with these perfusion defects and chronic thromboembolic changes, with or without pulmonary hypertension at rest (8, 10).

### 9.2 Frequency and the effect of acute reperfusion

Persistent limitation is common. In the prospective ELOPE cohort, 46.6% of patients had a peak oxygen uptake below 80% of predicted one year after PE (6). In FOCUS, the two-year cumulative incidence of post-PE impairment was 16.0% and that of CTEPH 2.3%; post-PE impairment identified 15 of the 16 patients eventually diagnosed with CTEPH (7). A meta-analysis estimated the incidence of CTEPH at 0.56% in all-comers, 3.2% in survivors and 2.8% in survivors without major comorbidities (78), consistent with earlier prospective cohorts reporting 1.5% and 3.8% at two years (79, 80). Reperfusion has not been shown to prevent chronic sequelae: in the three-year follow-up of PEITHO, tenecteplase did not reduce CTEPH (2.1% vs. 3.2%), persistent dyspnea or functional limitation (36.0% vs. 30.1%), or residual pulmonary hypertension and RV dysfunction on echocardiography (48). Whether CDI modifies long-term outcomes is unknown, and the long-term data of HI-PEITHO and PE-TRACT are awaited (31).

### 9.3 Structured follow-up and screening algorithms

A visit at approximately three months is the clinical anchor of follow-up (1, 4, 9). The Post-VTE Functional Status (PVFS) scale and the disease-specific Pulmonary Embolism Quality of Life (PEmb-QoL) questionnaire allow standardized and serial assessment (81, 82). Routine imaging of asymptomatic patients and screening for CTEPH in asymptomatic survivors are not recommended (ESC Class III) (1), whereas symptomatic patients with mismatched perfusion defects beyond three months should be referred to a pulmonary hypertension/CTEPH center (ESC Class I) (1).

The ESC position paper proposes echocardiography for patients with persistent dyspnea, functional limitation or CTEPH risk factors, followed by V/Q scintigraphy and referral when pulmonary hypertension is probable (4). In the InShape II management study (424 patients), an algorithm combining the CTEPH prediction score with electrocardiography and N-terminal pro-B-type natriuretic peptide (NT-proBNP) excluded CTEPH without echocardiography in 81% of patients, with a failure rate of 0.29% during two years, and identified 10 of the 13 CTEPH cases within four months (83). In InShape IV, adding a dedicated reading of the index CTPA for signs of chronic thromboembolism reduced the failure rate to 0%, with 20%–24% of patients requiring echocardiography (84), underlining the value of handing forward acute-phase imaging findings. A recent Delphi study among 39 European experts reached consensus that the three-month visit should assess the full spectrum of sequelae (CTEPH, CTEPD and post-PE syndrome) using patient-reported outcomes, that CPET is useful in persistent dyspnea, that right-heart catheterization belongs in specialized centers, that patients with persistent dyspnea need extended follow-up even after CTEPH and CTEPD are excluded, and that a single uniform protocol is preferable (85, 86). Our pathway integrating these elements is shown in Figure 2; its screening components rest on prospective management studies, whereas visit timing and multidisciplinary organization rest on expert consensus (4, 85).

**Figure 2:**
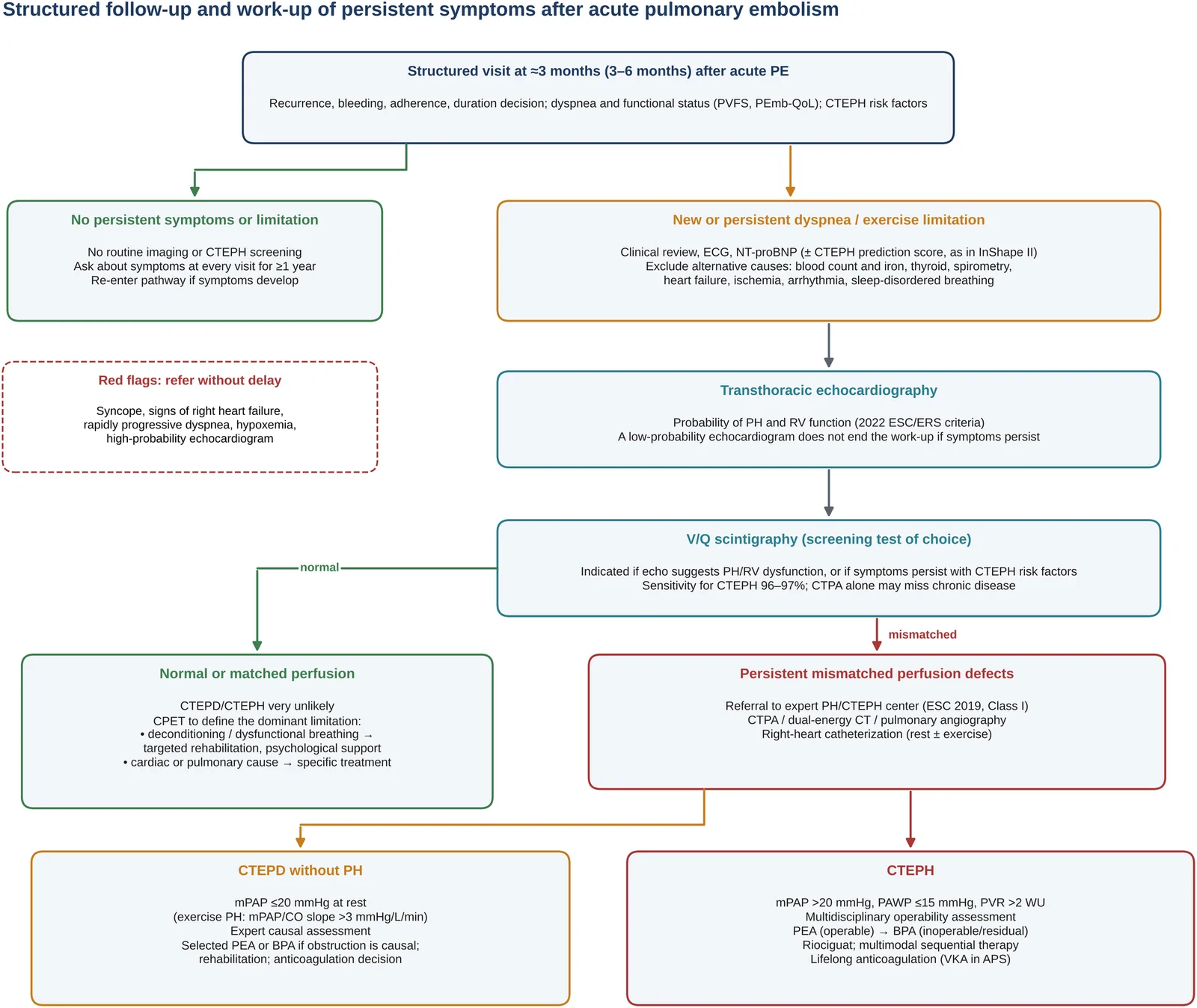
Proposed structured follow-up and diagnostic pathway for persistent symptoms after acute pulmonary embolism. The pathway integrates the 2019 ESC guidelines, the 2022 ESC position paper on follow-up, the 2022 ESC/ERS pulmonary hypertension guidelines, the 2026 AHA/ACC guideline, the 2025 European Delphi consensus on follow-up and the InShape II, InShape IV and FOCUS studies. Hemodynamic thresholds follow the 2022 ESC/ERS definitions. AHA/ACC, American Heart Association/American College of Cardiology; APS, antiphospholipid syndrome; BPA, balloon pulmonary angioplasty; CO, cardiac output; CPET, cardiopulmonary exercise testing; CT, computed tomography; CTEPD, chronic thromboembolic pulmonary disease; CTEPH, chronic thromboembolic pulmonary hypertension; CTPA, computed tomography pulmonary angiography; ECG, electrocardiography; ERS, European Respiratory Society; ESC, European Society of Cardiology; mPAP, mean pulmonary arterial pressure; NT-proBNP, N-terminal pro-B-type natriuretic peptide; PAWP, pulmonary arterial wedge pressure; PE, pulmonary embolism; PEA, pulmonary endarterectomy; PEmb-QoL, Pulmonary Embolism Quality of Life questionnaire; PH, pulmonary hypertension; PVFS, Post-VTE functional status scale; PVR, pulmonary vascular resistance; RV, right ventricular; VKA, vitamin K antagonist; V/Q, ventilation/perfusion; WU, Wood units.

### 9.4 Diagnostic tools for persistent symptoms

Transthoracic echocardiography is the usual first cardiac test when pulmonary hypertension or persistent RV dysfunction is suspected, but a low-probability echocardiogram does not end the investigation when symptoms are substantial (4, 10). V/Q scintigraphy is the preferred screening test for chronic thromboembolic obstruction: in a referral cohort, its sensitivity for CTEPH was 96%–97%, compared with 51% for CTPA at the time (87), and a normal perfusion scan makes CTEPH very unlikely (10). CTPA characterizes chronic thromboembolic lesions, but subtle disease is easily missed outside expert centers (8). Cardiopulmonary exercise testing (CPET) can distinguish deconditioning, ventilatory inefficiency, circulatory limitation, dysfunctional breathing and pulmonary vascular physiology; low end-tidal carbon dioxide and a high ventilatory equivalent for carbon dioxide suggest pulmonary vascular limitation (4, 10). Right-heart catheterization is required to confirm pulmonary hypertension and define hemodynamics before treatment (10).

The differential diagnosis of persistent dyspnea includes heart failure, ischemic and valvular disease, arrhythmia, chronic lung disease, anemia or iron deficiency, thyroid disease, obesity, sleep-disordered breathing, deconditioning, dysfunctional breathing and anxiety (4). Repeating CTPA without a specific hypothesis often detects residual material that does not establish causality; a staged evaluation (history, examination, electrocardiography, blood count, NT-proBNP and echocardiography, followed by targeted pulmonary function tests, V/Q scintigraphy or CPET) is more informative, and abrupt new breathlessness should be investigated for recurrence with comparison to baseline imaging (1, 4).

### 9.5 Rehabilitation

Mechanisms often coexist, and treating residual perfusion defects while ignoring deconditioning will not restore function. The evidence for rehabilitation consists of small randomized trials with heterogeneous populations and inconsistent results. Early-initiation exercise training was safe and improved fitness in a pilot randomized trial (88), and structured inpatient rehabilitation was feasible and safe (89). In a randomized trial of 140 unselected patients, a physiotherapist-guided home-based program did not significantly improve physical capacity compared with usual care (90), whereas in a randomized trial of 211 patients with persistent dyspnea 6–72 months after PE, eight weeks of supervised exercise training improved disease-specific quality of life and incremental shuttle walk distance (91). Rehabilitation should therefore be targeted to symptomatic patients after exclusion of CTEPH rather than offered indiscriminately. The AHA/ACC guideline considers early ambulation rather than bed rest reasonable (Class 2a) (9); in the authors' opinion, addressing fear of recurrence is an integral part of recovery.

### 9.6 Treatment of CTEPH and CTEPD

Suspected CTEPH should be evaluated by a multidisciplinary CTEPH team before any patient is declared inoperable (8, 10). Pulmonary endarterectomy is the potentially curative treatment of choice for operable disease (ESC/ERS Class I); in an international prospective registry, three-year survival was 89% in operated and 70% in non-operated patients (92), and in-hospital mortality has fallen to approximately 2% at highly experienced centers (93). Balloon pulmonary angioplasty (BPA) is recommended for technically inoperable disease or residual pulmonary hypertension after surgery (Class I) (10). In the RACE trial, BPA reduced PVR by approximately 60% versus 32% with riociguat at 26 weeks, at the cost of more procedure-related serious adverse events (94), and the MR BPA trial similarly showed greater hemodynamic improvement with BPA than with riociguat (95). Both trials support a sequential approach in which medical therapy precedes BPA in selected patients (10, 94).

Riociguat is recommended for inoperable or persistent/recurrent CTEPH (Class I); in CHEST-1, it improved six-minute walk distance by 46 m versus placebo, and PVR decreased by 226 dyn·s·cm⁻⁵ with riociguat versus an increase of 23 dyn·s·cm⁻⁵ with placebo (96). Subcutaneous treprostinil improved six-minute walk distance in severe non-operable CTEPH in the CTREPH trial (Class IIb) (97). Macitentan improved PVR in the phase 2 MERIT-1 trial, with sustained effects in its open-label extension (98), but the phase 3 MACiTEPH trial of macitentan 75 mg was stopped for futility on its primary endpoint of six-minute walk distance (99); its role therefore remains limited. Lifelong anticoagulation is recommended in CTEPH, with VKAs preferred in antiphospholipid syndrome (10). In symptomatic CTEPD without resting pulmonary hypertension, pulmonary endarterectomy improved symptoms and exercise capacity in selected patients at an experienced center (100), but this evidence is limited to single-center observational series, so intervention requires careful confirmation that obstruction is causally important and should be decided in expert centers (8, 10).

## 10 Implementation in real-world clinical systems

### 10.1 From PERT activation to longitudinal care

A mature PE program connects acute and outpatient care: the acute team records diagnostic certainty, provoking factors, RV and biomarker findings, treatment, complications, the intended anticoagulant course and follow-up triggers, and the outpatient team receives this information directly (4, 101). Standardized referral matters after advanced reperfusion too, because reperfusion has not been shown to prevent chronic sequelae (48). The AHA/ACC guideline considers follow-up in a specialized PE clinic reasonable for complex patients (Class 2a) (9). In the authors' opinion, a tiered model can return uncomplicated low-risk patients to primary or general care with a clear protocol, while recurrent, cancer-associated, pregnancy-associated, bleeding-prone, reperfused or persistently symptomatic patients receive specialist review.

### 10.2 Episode-wide quality indicators

We propose quality indicators covering the complete episode of care [authors' proposal, derived from guideline recommendations (1, 4, 9)]: (i) appropriate probability-based diagnosis; (ii) time to anticoagulation; (iii) documented risk category, including lactate in AHA/ACC categories C–E; (iv) correct discharge dose and transition schedule; (v) outpatient-eligibility assessment in low-risk patients; (vi) a documented provisional duration; (vii) completion of contact within one week; (viii) completion of a three-month review with standardized functional assessment (for example, PVFS); (ix) bleeding and recurrence at three and twelve months; (x) time from persistent symptoms to V/Q scintigraphy; and (xi) time to expert CTEPH referral. Patient-reported experience should be included, and the indicators require validation.

## 11 Critical appraisal, evidence gaps and research priorities

The evidence underpinning the recommendations discussed in this review is heterogeneous, and recommendations supported by randomized trials, prospective cohorts, device registries and expert consensus should not be presented with equivalent certainty. Table 10 therefore summarizes, for each key recommendation, the type of evidence available and the resulting level of certainty. Evidence for anticoagulation is robust, but important contemporary questions rest on weaker foundations. Until recently, CDI studies prioritized the RV/LV ratio at 24–48 h, enrolled selected patients and lacked randomized controls; observational comparisons are vulnerable to confounding by indication because the sickest patients are more likely to receive advanced treatment (11, 58). HI-PEITHO has narrowed but not closed this gap, because its benefit was confined to early physiological deterioration in a highly selected population (31). No randomized trial has compared MT with local thrombolysis in high-risk PE or tested any CDI against anticoagulation for long-term functional outcomes.

**Table 10:** Type of evidence and level of certainty for key recommendations discussed in this review.

Recommendation	Principal supporting evidence	Evidence type	Certainty
Probability-based diagnosis with D-dimer (age-adjusted, YEARS)	Prospective management studies (18, 19, 21); cluster RCT for PERC (20)	Prospective management studies; RCT	High
DOACs preferred over VKA in most patients	Phase 3 RCTs and meta-analysis (36)	RCTs, meta-analysis	High
Outpatient treatment of selected low-risk PE	RCTs and management studies (67–69)	RCTs, management studies	High
Systemic thrombolysis in high-risk PE	Small RCTs and meta-analysis (47); guideline consensus	Small RCTs, meta-analysis	Moderate
No routine systemic thrombolysis in normotensive PE	Large RCT with long-term follow-up (32, 48)	RCT	High
CDI in intermediate-high-risk PE with physiological stress	One RCT with early clinical endpoint (31); surrogate-endpoint RCTs (59, 61)	RCT (single), surrogate RCTs	Moderate (early outcome); low (mortality, long-term)
Mechanical thrombectomy versus CDT	One RCT with hierarchical, resource-driven endpoint (60)	RCT	Moderate
MT or local thrombolysis when systemic lysis is contraindicated (high-risk PE)	Registries and non-randomized studies (55, 58); consensus (11, 12)	Registry, expert consensus	Low
Surgical embolectomy; VA-ECMO	Observational series (35, 63)	Observational	Low
Reperfusion in normotensive shock (category D)	Retrospective registry analyses (33); guideline Class 2b (9)	Observational, expert consensus	Low
PERT evaluation	Before-and-after studies, meta-analysis (64, 65)	Observational	Low
Extended (reduced-dose) anticoagulation	RCTs (72–75)	RCTs	High
Minimum discharge bundle; contact within 1 week	Guideline recommendation (9)	Expert consensus, authors' proposal	Low
Structured 3-month review and CTEPH screening algorithm	Prospective cohort and management studies (7, 83, 84); Delphi consensus (85)	Prospective studies, consensus	Moderate
Rehabilitation for persistent symptoms	Small RCTs with inconsistent results (88, 90, 91)	Small RCTs	Low to moderate
PEA for operable CTEPH; BPA or riociguat for inoperable CTEPH	Registry (92); RCTs (94–96)	Registry, RCTs	Moderate to high
Intervention for CTEPD without resting PH	Single-center observational series (100)	Observational, expert consensus	Very low

Certainty was graded qualitatively by the authors: high, consistent evidence from randomized trials or large prospective management studies; moderate, a single randomized trial, randomized trials with surrogate or composite endpoints, or consistent prospective cohort data; low, observational or registry data or expert consensus; very low, small single-center series. BPA, balloon pulmonary angioplasty; CDI, catheter-directed intervention; CDT, catheter-directed thrombolysis; CTEPD, chronic thromboembolic pulmonary disease; CTEPH, chronic thromboembolic pulmonary hypertension; DOAC, direct oral anticoagulant; MT, mechanical thrombectomy; PE, pulmonary embolism; PEA, pulmonary endarterectomy; PERC, pulmonary embolism rule-out criteria; PERT, pulmonary embolism response team; PH, pulmonary hypertension; RCT, randomized controlled trial; VA-ECMO, venoarterial extracorporeal membrane oxygenation; VKA, vitamin K antagonist.

Long-term research is limited by inconsistent, overlapping definitions of post-PE syndrome, post-PE impairment, CTEPD and post-PE cardiac impairment, so prevalence depends on the definition used and on which patients return for assessment (5, 7, 8). Future trials need core outcomes encompassing survival, recurrence, major bleeding, hemodynamic deterioration, functional recovery, quality of life and diagnostic delay (4). Further priorities include validated dynamic risk models that do not multiply categories without changing treatment, phenotype-targeted rehabilitation trials, evaluation of whether systematic follow-up clinics improve time to CTEPH diagnosis and cost-effectiveness (102), and the inclusion of women, pregnant patients, older and frail adults, patients with cancer or renal dysfunction, and socioeconomically disadvantaged groups in PE trials (Table 11).

**Table 11:** Priority research questions and ongoing trials.

Area	Key question	Ongoing or recent studies	Preferred outcome
Reduced-dose systemic thrombolysis	Does reduced-dose alteplase improve net clinical benefit in intermediate-high-risk PE?	PEITHO-3 (50)	Death, decompensation, major and intracranial bleeding
Normotensive shock (AHA/ACC category D)	Can incipient cardiopulmonary failure be identified prospectively, and does targeted treatment improve outcomes?	Retrospective registry data only (33, 34)	Prospective validation; mortality; decompensation
CDI versus anticoagulation	Which patients derive net benefit from early CDI, and does it improve long-term function?	HI-PEITHO long-term follow-up (31); PEERLESS II; PE-TRACT	Patient-important outcomes; functional capacity; quality of life
High-risk PE with lytic contraindication	Mechanical thrombectomy versus local thrombolysis or surgery	No randomized trial	Mortality, bleeding, recovery
Post-PE syndrome	Which phenotype benefits from rehabilitation or intervention?	Rehabilitation RCTs (90, 91)	Exercise capacity; quality of life
CTEPH screening	Can structured algorithms shorten diagnostic delay efficiently?	InShape II (83); FOCUS (7)	Time to diagnosis; operability; cost
Extended prevention	How should recurrence and bleeding risk be updated over time?	RENOVE (74); API-CAT (75)	Net clinical benefit; patient preference
Care delivery	Do integrated PERT-to-clinic programs improve outcomes and equity?	Observational data (64, 65)	Continuity, safety, equity, patient experience

CDI, catheter-directed intervention; CTEPH, chronic thromboembolic pulmonary hypertension; PE, pulmonary embolism; PERT, pulmonary embolism response team; RCT, randomized controlled trial.

## 12 Conclusions

PE care should be designed as one longitudinal episode (Figure 1). The ESC and AHA/ACC frameworks differ in terminology but converge on the principles that hemodynamics defines urgency, that RV dysfunction, myocardial injury, lactate and clinical trajectory refine prognosis in normotensive patients, and that anticoagulation—preferably with a DOAC—is the foundation of treatment. Systemic thrombolysis remains reserved for high-risk (category E) PE. HI-PEITHO provides the first randomized evidence that CDI reduces early decompensation in carefully selected intermediate-high-risk patients, but survival and long-term functional benefits remain unproven, and MT is the preferred CDI when fibrinolysis is absolutely contraindicated. The discharge process must convert a successful acute intervention into a safe recovery plan, with early contact and a structured three-month review. Persistent symptoms warrant phenotype-directed evaluation rather than automatic repeat CTPA, with V/Q scintigraphy as the screening test for chronic thromboembolic disease, hemodynamic confirmation using the 2022 thresholds and early referral to an expert center, where pulmonary endarterectomy, BPA and medical therapy can be combined.

## Supplementary material

SUPPLEMENTARY TABLE S1: Full search strings used for the literature search (PubMed/MEDLINE).
