# Supplementary material for: Integrated management of acute pulmonary embolism and the post-pulmonary embolism syndrome: risk stratification, modern therapies, and long-term follow-up
Source: Front Cardiovasc Med. 2026 Oct 21;13:2002611. doi: 10.3389/fcvm.2026.2002611 (PMC13732469; doi:10.3389/fcvm.2026.2002611)
Supplement: Supplementary file 1 [file Table1.docx]

**Supplementary Material**

*Integrated Management of Acute Pulmonary Embolism and the Post-Pulmonary Embolism Syndrome: Risk Stratification, Modern Therapies, and Long-Term Follow-Up*

**Supplementary Table S1. Literature search strategy.**

| **#** | **Domain** | **PubMed/MEDLINE search string** |
| --- | --- | --- |
| 1 | Core concept | "Pulmonary Embolism"[MeSH] OR "pulmonary embolism"[tiab] OR "venous thromboembolism"[tiab] |
| 2 | Diagnosis and risk stratification | #1 AND ("D-dimer"[tiab] OR "clinical decision rule*"[tiab] OR YEARS[tiab] OR "rule-out criteria"[tiab] OR "computed tomography pulmonary angiography"[tiab] OR "ventilation perfusion"[tiab] OR "risk stratification"[tiab] OR "severity index"[tiab] OR troponin[tiab] OR "right ventricular dysfunction"[tiab] OR lactate[tiab] OR "normotensive shock"[tiab] OR "cardiogenic shock"[tiab]) |
| 3 | Anticoagulation and secondary prevention | #1 AND ("direct oral anticoagulant*"[tiab] OR apixaban[tiab] OR rivaroxaban[tiab] OR edoxaban[tiab] OR dabigatran[tiab] OR "low molecular weight heparin"[tiab] OR "extended treatment"[tiab] OR "reduced dose"[tiab] OR "outpatient treatment"[tiab] OR "early discharge"[tiab]) |
| 4 | Reperfusion and advanced therapy | #1 AND (thrombolysis[tiab] OR fibrinolysis[tiab] OR alteplase[tiab] OR tenecteplase[tiab] OR "catheter-directed"[tiab] OR "ultrasound-facilitated"[tiab] OR "ultrasound-assisted"[tiab] OR "mechanical thrombectomy"[tiab] OR "aspiration thrombectomy"[tiab] OR "large-bore"[tiab] OR embolectomy[tiab] OR "extracorporeal membrane oxygenation"[tiab] OR "response team"[tiab] OR "vena cava filter*"[tiab]) |
| 5 | Follow-up and post-PE syndrome | #1 AND ("post-pulmonary embolism syndrome"[tiab] OR "post-PE syndrome"[tiab] OR "post-PE impairment"[tiab] OR "functional limitation*"[tiab] OR "functional status"[tiab] OR "quality of life"[tiab] OR "cardiopulmonary exercise"[tiab] OR rehabilitation[tiab] OR "exercise training"[tiab] OR "follow-up"[tiab] OR "chronic thromboembolic"[tiab]) |
| 6 | CTEPD and CTEPH | ("chronic thromboembolic pulmonary hypertension"[tiab] OR "chronic thromboembolic pulmonary disease"[tiab] OR CTEPH[tiab] OR CTEPD[tiab]) AND (incidence[tiab] OR screening[tiab] OR algorithm[tiab] OR endarterectomy[tiab] OR "balloon pulmonary angioplasty"[tiab] OR riociguat[tiab] OR macitentan[tiab] OR treprostinil[tiab]) |
| 7 | Filters applied to #2–#6 | English; humans; adults (≥18 years); publication date 2000/01/01–2026/08/31; article types: practice guideline, consensus/position statement, randomized controlled trial, meta-analysis, systematic review, prospective cohort, management study, registry |
| 8 | Trial and registry names (searched individually) | PEITHO; PEITHO-3; HI-PEITHO; ULTIMA; SEATTLE II; OPTALYSE; SUNSET sPE; CANARY; FLARE; EXTRACT-PE; FLASH; FLAME; PEERLESS; PEERLESS II; STORM-PE; PE-TRACT; OTPE; HoT-PE; HOME-PE; COBRRA; AMPLIFY-EXT; EINSTEIN CHOICE; RENOVE; API-CAT; ELOPE; FOCUS; InShape II; InShape IV; CHEST-1; MERIT; MACiTEPH; CTREPH; RACE; MR BPA |
| 9 | Other sources | Websites of the ESC, ERS, AHA/ACC, American College of Chest Physicians, American Society of Hematology and International Society on Thrombosis and Haemostasis (guidelines and consensus documents); ClinicalTrials.gov ("pulmonary embolism" AND [thrombolysis OR thrombectomy OR catheter-directed], status recruiting or active, not recruiting); forward citation tracking of the 2026 AHA/ACC guideline and the 2022 ESC position paper on follow-up; reference lists of included guidelines and key reviews |
| 10 | Exclusions | Case reports and small case series (except where no other evidence existed), conference abstracts, unpublished data, pediatric studies and non-English publications |

The search was first performed during manuscript preparation and repeated during revision with the addition of trial-name searches (row 8) and forward citation tracking (row 9). Records were screened on title and abstract and then on full text against the eligibility criteria described in the Methods. Abbreviations: AHA/ACC, American Heart Association/American College of Cardiology; CTEPD, chronic thromboembolic pulmonary disease; CTEPH, chronic thromboembolic pulmonary hypertension; ERS, European Respiratory Society; ESC, European Society of Cardiology; MeSH, Medical Subject Headings; PE, pulmonary embolism; tiab, title/abstract.
